# Meeting Men Where they are: Motivators and Barriers to Accessing Health Services through a Men’s Mobile Wellness Clinic, October 2019 to March 2020, Blantyre, Malawi

**DOI:** 10.1007/s44197-024-00306-5

**Published:** 2024-10-10

**Authors:** Mtemwa Nyangulu, Tiffiany Aholou, Viva Thorsen, Shahul Ebrahim, Ernest Nkhoma, Chipiliro Payesa, Getrude Chipungu, Masozie Kalua, Jeroen van ’t Pad Bosch, Hannah Gibson, Verita Buie, Fidelis Sindani, Helen Dale, Stephanie Behel, Rashida Hassan, Alice Maida, Kristina Grabbe

**Affiliations:** 1 US Centers for Disease Control and Prevention, Lilongwe, Malawi; 2 Jhpiego, Lilongwe, Malawi; 3 Jhpiego, Baltimore, MD USA; 4 Division of Global HIV and TB, Centers for Disease Control and Prevention, Atlanta, USA

**Keywords:** HIV Testing Services, Men, Mobile Clinic, Employer, Workplace, Health Services, Malawi

## Abstract

**Background:**

In Malawi approximately, 88.3% people living with HIV are aware of their HIV status. Significant gaps are among men aged 15–34 years; only 72% know their HIV status. To reach men, Jhpiego, in collaboration with the Ministry of Health (MOH), implemented the Men’s Mobile Wellness Clinic (MMWC) at workplace settings in Blantyre, Malawi between October 2019 and March 2020.

**Methods:**

We conducted a descriptive qualitative study to understand motivators and barriers to MMWC service uptake by employees and employers. Primary data was drawn from in-depth telephone interviews from four study populations: employers who accepted or declined to host the MMWC at their worksite, and employees who accessed or did not access the services. We performed a thematic analysis using Nvivo 12 software to identify patterns and themes across the dataset.

**Findings:**

Main reasons given for using the service among male employees were a desire to know their health status, availability of free health services at the workplace, and good quality services offered by MMWC staff, and support from their supervisor. Men who did not access services stated reasons such as work-clinic scheduling conflicts, lack of adequate promotion of the service, and miscommunication on the criteria about who should attend the MMWC. Employers who accepted to host the MMWC stated convenience and employee’s rights to know their health status. Those who declined either stated that employees did not want the services or COVID-19 preventive measures by the MOH between October 2019 and March 2020 restricted participation.

**Conclusion:**

This study underscores the potential utility of MMWC services including HIV testing among men. The desire to know their health status, availability of free MMWC services at the workplace, good quality services offered by MMWC staff, and the endorsement of MMWC by supervisors were main motivators to access the MMWC services. Sensitizing supervisors and employees about the benefits of the MMWC services, strengthening demand creation, and clarifying eligibility are important to facilitate MMWC uptake among men in Malawi.

## Background

In Malawi 1,013,261 people were living with HIV (PLHIV) in 2022, with the majority (87,470) living in Blantyre City [1]. It is estimated that 88.3% of PLHIV are aware of their HIV status, yet only 72% of men aged 15–34 years are aware of their status, and 35% have never tested for HIV [2]. In multiple countries such as Malawi, Lesotho, Mozambique, and South Africa, evidence-based approaches on the common barriers to HIV health seeking behavior for men are summarized on the President’s Emergency Plan for AIDS Relief (PEPFAR) Solutions Platform. Inaccessible hours of HIV services, most of which are during the working day, facility location and setup, and insufficient education about the importance of knowing one’s HIV status may present barriers to access for men [3, 4]. As in other countries in Sub-Saharan Africa, women are disproportionately affected by HIV [5]. However, among men who are HIV-positive, compared to women, a lower proportion know their status, are on antiretroviral therapy (ART), and are virally suppressed [2]. Men represent an important group to engage in HIV testing and prevention services because of the prevailing cultural and gender norms in Malawi. Some studies have reported that poor health-seeking behavior by men may be associated with the patriarchal culture, where men in Malawi are unlikely to seek care, either as a guardian or as a patient [5]. Other known barriers to access include masculinity beliefs, work commitments, and difficulties accessing conventional primary health care without appointments [6]. Innovative HIV testing services are needed to increase awareness of HIV status and access to treatment, especially among hard-to-reach populations [7]. Since 2000, Malawi HIV testing services (HTS) have been offered by trained non-laboratory personnel to increase coverage and are free of charge to individuals at all Ministry of Health (MOH) certified health facilities including respective community HTS sites [8]. Despite the availability of free HIV testing services, there has been a low uptake of HIV testing services among men in Malawi [4, 9].

Previous HIV testing studies show that community-based HIV testing and counselling (testing outside of health facilities) has the potential to reduce coverage gaps among men, but the relative impact of different modalities is not well assessed [10]. Studies aimed at reaching underserved populations include workplace HTS. The World Health Organization (WHO) recommends that workers in industries (military, mining, construction, security, petroleum, agriculture, fishing, long-distance driving, and many others) may not have easy access to HIV testing services, and the workplace may be the best place to reach them [11]. A study on men’s perceptions of drivers and barriers of workplace-based HIV self-testing in Uganda reported the involvement of employers as a driver to HIV testing. The barriers reported were the cost of HIV tests, stigma, lack of testing support, fear of discrimination and isolation, and concerns around decreased work productivity in the event of a reactive self-test [12]. A qualitative study on barriers and motivators of HIV testing among men in Kenya reported that barriers to testing included perceived providers attitudes, facility location and set up, wait time/inconvenient clinic times, low perception of risk, limited HIV knowledge, stigma, discrimination, and fear of having a test. High risk perception, severe illness, awareness of partner’s status, confidentiality, quality of services and supplies, flexible/extended opening hours, and pre–and post–test counselling were motivators [13]. To better address the gap in HIV awareness and improve HTS uptake among men at workplaces in Malawi, targeted HTS approaches are needed. The purpose of this study is threefold: Firstly, to explore the motivators and barriers influencing service uptake among male employees in workplace settings through the Men’s Mobile Wellness Clinic (MMWC). Secondly, to understand the factors that influenced employers in implementing the MMWC at the workplace. Lastly, to glean recommendations from employees and employers regarding service utilization at the Men’s Mobile Wellness Clinic (MMWC). The results of the study can be used to ensure equitable access to services that meet men’s unique needs and increase coverage of HIV testing services in high burden geographical areas.

## Methods

### Study Design Sites and Population

#### Study Overview

Jhpiego, a CDC community supported partner, implemented a Gateway Project which provided community-based HIV Testing Services through mobile and outreach services including moonlight testing, targeted testing using a mobile van and tents to access high HIV prevalence geographical areas, targeted home-based approaches to reach partners of PLHIV (index testing) and workplace testing. In collaboration with MOH, Jhpiego implemented the Men’s Mobile Wellness Clinic (MMWC) at 26 workplaces from October 2019 to March 2020 in Blantyre City, targeting men working in industrial sites hosting a variety of businesses including agro-processors, garment, pharmaceutical, and consumer goods manufacturers, as well as breweries and timber companies. The MMWC provided HIV testing; screening for non-communicable diseases including diabetes, hypertension, and obesity; lifestyle counseling; malaria screening; and syphilis testing services. A total of 1,242 men aged 18 years and above voluntarily utilized at least one clinic service. Linkage and referrals to other health services and treatment were provided as needed. The MMWC services were brought to workplaces during the daytime on weekdays for approximately 10 h, including lunch time. Individuals who consented for HTS were tested by trained and certified HIV Diagnostic Assistants.

#### Site Selection

Jhpiego, in collaboration with MOH, implemented a qualitative descriptive study in October 2021 to understand the motivators and barriers to service uptake. Telephone interviews were conducted with employers who accepted and declined the MMWC services at their workplace and employees who accessed or did not access MMWC services at workplaces. Jhpiego informed all workplaces through their respective leadership about the study including purpose, participant incentive (phone credit), and estimated amount of time. Letters describing scope of the study were sent to workplaces followed by physical meetings with leadership who sensitized the employees about the study. All eligible employees and employer representatives provided verbal consent to participate in the study through telephone interviews. Purposive sampling was employed when recruiting the workplaces as study sites. Twelve of the 26 sites were selected for inclusion based on the size and industry type to include a variety of participants, experiences, and responses. Companies were selected with different size ranges from less than 50 employees to over 100 employees. Industry types at these 12 sites included agribusiness, aviation, transport, food and beverage processing, cement manufacturing, timber companies, and textile company.

#### Recruitment of Study Participants

The study employee participants were grouped into two age groups for analysis: 18 to 30 years and above 30 years to determine any systematic differences between the age groups in their responses. The study team contacted participants using a list obtained from respective employer and agreed on a time and date to conduct the telephone interview. The study participants provided oral consent to participate in a telephone interview including audio recording.

### Inclusion and Exclusion Criteria

Participants were men 18 years and above that were employed at the site during MMWC regardless of their use of MMWC services. The study employed other exclusion and inclusion criteria for the men to be sampled in the study population, detailed in Table 1. Employers who either accepted or declined the MMWC services at workplace participated in the study through a representative of one workplace managers.

**Table 1:** Summary of the inclusion and exclusion criteria for the men’s mobile wellness clinic study participants, Blantyre, 2020

Inclusion Criteria	High level managers from employers who accepted and declined the MMWC services and Employees at workplaces that accessed and did not access MMWC services: Employed at the site during mobile wellness clinic Eighteen years of age and above Able and willing to attend telephone interview Willing to provide a telephone number to participate in interviews Consent to participate in the study Agree to be audio-recorded as part of interview
Exclusion Criteria	Employees at the workplaces that accessed and did not access services: Not employed during the men’s mobile wellness clinic Under the age of 18 years Unable to attend telephone interview Unable to provide a phone number to confirm participation or Unwillingness to be audio-recorded

### Data Collection and Management

Due to COVID-19 prevention measures, telephone interviews were utilized to collect data using a semi-structured interview guide (Table 2) aligned with study objectives and research questions. The tools were developed in English and translated into Chichewa, the primary local language in Malawi. Back translation was not done; however, study staff reviewed interview guides for concurrence to ensure the quality of translation. Four experienced research assistants were trained in interviewing and oriented on study protocol including data collection tools. There were two research assistants per interview session, which lasted approximately 30 min. One was responsible for interviews and the other wrote notes in addition to audio recording. The Principal Investigator and Study Coordinator provided daily oversight to ensure adherence to study protocols. The research assistants used the semi-structured interview guide to conduct the interviews in either English or Chichewa depending on the participant’s language preference, to ensure both could understand each other well. The interview sessions were recorded, and the audio files were stored in a password-protected computer. The interviews were transcribed verbatim for analysis. We ensured confidentiality by omitting identifying information and assigning unique identification numbers to each participant and storing all their data securely. The study participants’ details were stored in a secured, lockable place for access only by the research team.

**Table 2:** Sample of prompts used in employee and employer interview guide for men who participated in MMWC study, Blantyre, 2020

**Employee Interview Guide**
Let’s start by discussing how, when and where did you first hear about the mobile wellness clinic? [Probe: How did they hear about it? How was the clinic advertised? Where did they hear about it? ]
How were the dates and hours of the mobile wellness clinic advertised? How were the dates and hours convenient for you?
Why did you accept services from the Mobile Wellness Clinic? [Probe: type of service, convenience, cost/no cost, confidentiality etc.]
Which services did you use but did not like or find valuable and why? [Probe: Describe.]
Which services were not offered that you wish were offered? [Probe: please explain why you would have liked these services]
What did you think of the quality and variety of services offered?
What did you think of the knowledge and skills of the service providers who attended to you?
Did you feel supported by your employer to attend the mobile wellness clinic? [Probe: Why or why not? Describe]
What recommendations do you have to improve quality of service? To improve service uptake? [Probe: wait times, length of service, type of service provider, privacy, return of results, follow up, different location. Also ask if there are any specifically about HIVST.]
What do you perceive to be the challenges with reaching men with HIV testing services at the workplace or in the community?
What is your opinion on the mobile wellness clinic in terms of its ability to serve men at the workplace/in the community, especially for HIV testing?
Would you recommend the mobile wellness clinic to others? Why or why not?
Would you return to the mobile wellness clinic in the future? Why or why not?
**Employer Interview Guide – declined services on site **
Is there currently or was there an employer sponsored clinic on the premises in 2019/2020? If so, please describe the services, attendance trends, etc. [Probe: When – what month; duration; hours of operation; services; target population; feedback from staff if any’ attendance trends]
What factors led to declining the mobile wellness services at this location?
Who was involved in the decision to decline the mobile wellness services? [Probe: how many people? what were their roles? What was the process to make the decision]
What are the perceived benefits to mobile wellness clinics at the workplace? [Probe: What are your thoughts about offering these services to men in this community or at the workplace? )
Under what circumstances would the company accept the mobile wellness clinic services at the workplace? If you were to accept services in the future, what is the preferred frequency? [Probe: month/days/duration]
What additional services, if any, you would like to see offered by the mobile clinic that would lead to accepting the service at the worksite? [interviewer may need to list the services currently offered by mobile wellness clinic]
What do you believe discourages service uptake by men at the workplace? What discourages the uptake of HIV testing by men?
What recommendations do you have on how to increase acceptability and service uptake by men, especially HIV testing?

### Data Analysis

The analysis was conducted in two phases. First, the project coordinator/researcher (PC/R) used NVivo 12 for data organization and analysis following transcription and translation of the audio interviews. Next, the codes were developed based on the objectives of the study, research questions of interest, and reviewing the transcripts. The coding framework gave the researcher directions and steps to identify themes, using a thematic content analysis to analyze all data categories from interviews. This involved reading transcripts multiple times to understand the data in context and attaching labels to excerpts based on meanings that the researcher discerned. Sections of text were conceptualized by constant comparison of points raised by different people within the interviews. In phase two of analysis, a separate research team made up of HTS subject matter experts versed in qualitative research used phase one themes as an initial basis to read through a subset of transcripts [36] to validate and refine themes and subthemes from the original findings. The research team met to discuss discrepancies until consensus was reached.

### Ethical Considerations

This activity was reviewed by CDC, deemed not research, and was conducted consistent with applicable federal law and CDC policy. Likewise, Johns Hopkins University in the US (FWA00003428) and the Malawi National Health Sciences Research Committee, a local institutional review board (FWA00005976 IRB00003905), both granted approval for this project.

## Results

We conducted seventy-three in-depth interviews (IDIs) via telephone with employees who accessed the men’s wellness clinic services, employees who did not access the services, employers who accepted the MMWC, and employers who declined the MMWC. The primary data collection for this study was conducted for one month (October 5, 2021 to November 5, 2021) at workplaces in Blantyre.

### Summary Characteristics of Participants

The participants in this study were categorized into two groups: employees and employers. The employees were categorized into 4 groups. Employees were disaggregated into four groups: men who accepted services between the ages 18–30 and men who accepted services over the age of 30; men who declined services between the ages 18–30 and men who declined services over the age of 30. About 51% of employees interviewed worked day shifts from 6am to 5pm while 49% worked at night. Men who worked full-time reported working 8–11 h per day, five or six days per week and indicate that this was the norm for most of the participants. Among the employees, the majority were over 30 years of age and worked fulltime. Eleven employers were interviewed to understand reasons for acceptance of the men’s wellness clinic services. The selection criterion for the employers was their involvement in the decision of accepting or declining the MMWC services and a willingness to be interviewed and audio recorded. Employers were grouped into whether their establishment allowed or did not allow the MMWC services (Tables 3 and 4). Pseudonyms and a code to denote the age group and whether the participant accessed (for employees) or accepted (for employers) the MMWC services as descriptors to excerpts to provide additional context.

**Table 3:** Study participation by employees and employers who accessed or did not access the Men’s Mobile Wellness Clinic services, Blantyre, 2020

Service Assessment Sample category	Code	Original Sample Size	Actual sample size
Men who accessed services (> 30 years of age)	A > 30	15	21
Men who accessed services (18–30 years of age)	A18-30	15	11
Men who did not access services (> 30 years of age)	DA > 30	15	17
Men who did not access services (18–30 years of age)	DA18-30	15	13
Employers who accepted services	EAS	8	8
Employers who declined services	EDS	4	3
Totals		72	73

**Table 4:** Characteristics of employees who participated in Men’s Mobile Wellness Clinic study, Blantyre, 2020

Characteristics	Accessed		Did not access	
Type of employment	18–30 years of age	> 30 years of age	18–30 years of age	> 30 years of age
Contract	3	8	2	3
Full time	8	13	11	14
Marital status				
Single/widowed	4	5	3	6
Married	7	16	10	11
Total	11	21	13	17

### Motivating Factors and Barriers to the Use of Men’S Mobile Wellness Clinic Services

#### Motivating Factors

Four sub-themes were identified as factors that influenced employees’ decision to attend the MMWC: desire to know their health status, availability of free MMWC services at the workplace, support from their supervisor and good quality services offered by MMWC staff.

##### Desire to Know Their Health Status

Most participants accepted the men’s mobile wellness services because they wanted to know their health status. Some were motivated to access the services because they were unsure of their HIV status due to some risky behaviors, while others wanted to be screened to know if they had other health problems.

“*Because sometimes you doubt yourself on how you have been behaving*,* so you just need to know how your health is*”- [Timve, A18-30 ].

“*I wanted them to provide to me all the services*,* which they were offering*,* because I have no time to visit the hospital. Apart from HIV*,* which I normally test for*,* I also wanted to test for BP and other diseases*,* which I have not tested for before.” -* [Chisomo, A > 30].

##### Availability of Free MMWC Services at the Workplace

Men in this study reported working long hours, and the nature of their work was not conducive to taking time off. The MMWC provided the employees with easy and convenient access to health services, including blood pressure checks, body mass index measurements, syphilis testing, and blood sugar testing, which they perceived as beneficial as these are not routinely offered for free at health facilities.

*“For me*,* I am always busy*,* and when they said a mobile clinic will be at our workplace*,* I was so happy. I don’t have time to go to the hospitals because I am always working”*- [Dalo, A > 30].

*“You know one cannot go to hospital and have these tests on demand at no cost; as to having to be sick and possibly paying for the service. Here they were for free*,* I was happy to check”-* [Sinya, A > 30].

##### Support From Their Supervisor

When asked whether the employees felt supported by their employer to access the MMWC, men, including some who did not access the services, responded positively placing emphasis on the support of their supervisor.

*“My employer encouraged us to attend the mobile clinic. I know not everyone attended the clinic but some of us showed interest because we were told by our employer. So*,* he really helped me because by now I wouldn’t have known my status on BP*,* sugar” - employee who accessed [*Goli, A > 30].

In contrast, there were a few instances where the employer made provisions for the MMWC to be at the workplace, yet the employees did not feel supported as expressed by an employee who did not access the services:

*“… we were busy but really wanted to go and we had the interest of going*,* but he [ the employer] did not support us. He kept us busy; we were still working.”* [Nyuma, DA18-30].

##### Perception of Good Quality Services Offered by MMWC Staff

When asked about the quality of services they received, most men who accessed the services expressed satisfaction. They used terms such as “easy,” “quick,” and “professional” to describe the process to request services, to receive services, and the quality of services by the staff, respectively. The privacy aspect of the services (one-on-one) and the counseling they received following receipt of results are other key aspects of the services that the men spoke favorably about. Coupling HIV testing with screening of other diseases/health conditions was another motivator for men to use the MMWC.

*“I got an HIV test*,* I got my weight checked on a scale*,* I got BP check*,* I tested for diabetes……. YES…….staff knowledge and skills were good because they were able to enlighten you on what to do; they explained all the important details to you”* – [Mphatso, A > 30].

*“Confidentiality was good when giving results it was only two of you the provider and the client*,* there was not any other person who could hear about your results”* – [Zika, A18-30].

#### Barriers to Accessing the Men’S Wellness Clinic Services

Three key factors were mentioned to have influenced employee’s inability to access the MMWC: mobile clinic hours did not align with work schedules, lack of adequate promotion, and miscommunication on criteria about who should attend the wellness clinic.

##### Mobile Clinic and Work Time Conflict

Most employees who did not access the MMWC services reported a conflict with their busy work schedule. For example, factory employees work in shifts, and the services were offered during their working hours. Some of the employees were on night duty when the MMWC services were offered, thereby making the MMWC services unavailable for them to access.

*“I did not refuse but that day I was working the night shift”-* [Chimwemwe, DA18-30].

*“It was not convenient with my work* [Chilungamo, DA > 30].

##### Lack of Adequate Promotion

A second stated reason for not accessing the MMWC was inadequate promotion of the MMWC services. Among men who accessed and who did not access services, most mentioned they learned about the MMWC on the day the services were offered. Among employees who did not access the MMWC, they reported that they were too busy to access the services:

*“I did not refuse but that day I was working*,* and I heard about the clinic too late”* [Bwino, DA > 30].

With same day promotion, some staff who worked night duty or were off had to rely on word of mouth - though this would often be too late:

*“ Because when one is working night shift*,* they can’t attend any HR meetings conducted during the day. So*,* we rely on our colleagues to relay the information to us*. [Chikondi, DA > 30].

##### Miscommunication on Criteria About Who Should Attend the Wellness Clinic

Although the MMWC did not include a criterion related to full-time, part-time, or contractors, some men reported that they missed the opportunity to attend the MMWC because there was miscommunication on staff criteria to access the clinic services. They were informed that the services were for permanent staff members and not for the staff on short-term contracts.

*“We were not told anything because they told us that as contractors*,* we are not supposed to do the testing but only staff members*,* so we didn’t pay attention to ask what it was all about*.”- [Mbweno, DA > 30].

##### Other Reasons Why Men Chose Not to Access MMWC Services

When asked to explain why some men did not access the MMWC services, a few rare, yet noteworthy reasons included low risk perception, fear of stigma and discrimination of receiving a positive HIV test result at the workplace, and having received a recent HIV negative test result.

*“Aaaa I refused since am a person who doesn’t get sick… Yes*,* but the only thing is that I told them that I don’t have a problem*,* because I don’t get sick.”* [Yamikani, DA18-30.

*“Because that time it was my first time going for a blood test*,* so you know what happens*,* I had pressure and I was stressed that maybe I will test positive”* [Dziko, DA > 30].

“*aaah in terms of visiting their workplaces*,* let’s say you have tested positive*,* some people do not think properly once they know that you are positive. They start discriminating and treating you differently*,* that’s what I can say.” [Madalitso*,* DA18-30].*

### Drivers to Employer’S Acceptability and Decline of MMWC Services in the Workplace

Convenience and employee’s right to know their status influenced employers’ decision to accept MMWC services. The employers who accepted the services stated that the MMWC at workplace was convenient as employees are often too busy to attend traditional clinics. The employers were mindful that employees have rights to access health services and know their status.

*“After seeing that we could not provide some services*,* we thought that if they come and provide such services*,* we saw it wise that aaah it would be better for them to come and provide those services to see how it could* be?” – Manager who accepted. [Dolo, EAS].

Two workplaces did not accept the MMWC services. One manager stated that COVID-19 prevention restrictions was the main reason for not accepting the services. Government preventive measures stopped gatherings and meetings hence people from outside managing the MMWC services were not allowed to offer the services. The other manager reported that employees themselves were consulted and stated they did not want the services.

*“It was because of COVID-19 and there were restrictions in gatherings and meetings*,* so it was felt we should not be having unknown people from outside”.* [Kondwa, EDS].

*“So*,* we are afraid of wasting peoples time*,* because we can accept that you should come with the mobile clinic*,* and it may happen that nobody is interested to attend*,* So before that we always have a meeting with the employees*,* So that we can hear their thoughts*,* so after we called them almost*,* starting with the leaders who met them asking everyone questions*,* it happened that no one was interested”*- [Zakeyo, EDS].

## Participants and Employers’ Recommendations

Table 5 summarizes the main recommendations made by employees and their respective employers on how best to improve MMWC service and increase their uptake among men. Other recommendations included provision of male-friendly services; offer other services like male circumcision; sensitizing employees to understand the benefits of services with an emphasis on privacy / confidentiality; improving demand creation of the MMWC; providing promotional incentives; offering services at various times of the day and across multiple days; addressing misconceptions about MMWC services; and incentivizing HTS.

**Table 5:** Employees and their respective employers MMWC services recommendations, Blantyre, 2020

Recommendation	Quote
Sensitize employees to understand the benefits of receiving the services offered with an emphasis on privacy / confidentiality.	*“When you explain to them*,* they understand the services*,* then they are willing that MMWC should come*,* they will attend*,*”* (EAS)
Improve demand creation of the MMWC	*“It’s also necessary to inform the people in advance about the services which you will offer*,* the dates etc. to prepare them psychologically.”* *“……and another thing is probably to highlight the advantages of going for testing”*(EDS)
Men prefer to receive services separate from women	*RESPONDENT: “maybe you should be coming on two mobile clinics one for women one for men”* *INTEVIEWER: “okay so your opinion is to separate you from women?”* *RESPONDENT: (laughs) “yes”*(EDS)
Offer the services at various times of the day and increase the number of days (e.g., for a full week) to reach those who work on shifts or who may have been off duty	*“It would be good if it would be coming for an entire week*,* Monday to Friday so that it caters for a lot of people so that those you can be able to find those that were working day and night shifts but also those that had taken their off days. I ……… I think that it is a good thing that you need to do frequently because men find it hard to go to the hospital”* (EAS)
Offer other services	*“Giving us counselling on how to prevent this general disease but not only HIV but different illnesses*,* giving counselling on what to prevent and so on and so forth”* (EDS)
Confidentiality	“*I would say number one you have to clear that misconception that management may come to know the results or status”*(EDS)
Consider providing promotional incentives (e.g., t-shirts)	*‘Incentivize the activity or the initiative it could be through freebies or gifts*,* it would not have to be monetary but just probably some t-shirts*,* some caps*,* branded ones and you can collaborate with our company probably and you can brand the t-shirt with both logos.”* *“…so*,* I have mentioned two*,* incentivizing and clearing the misconception*,* …”* (EAS)

## Discussion

The Men’s Mobile Wellness Clinic (MMWC) in Blantyre City, Malawi, aimed to address the gap in HIV awareness and improve the uptake of HTS among men in workplace settings. Our study revealed that the primary motivation for men in industrial sectors to seek MMWC services was the desire to know their health status. This finding aligns with existing literature that emphasizes the importance of health awareness in promoting HIV service uptake [3].

Previous studies have highlighted various access barriers faced by men when seeking HIV and other health services. These barriers include high costs (such as transportation expenses and time off work), long wait times at traditional clinics, gendered health systems that lack male-friendly services, and provider biases [14]. In our study, employees who accepted the MMWC were motivated to access services because they were free, convenient, and quality services offered directly on their workplace premises. This approach demonstrated corporate social responsibility, whereby businesses seek to create a win-win for themselves and the community by investing in the well-being of their employees and underscored their commitment to employee health and well-being [15]. Meeting men where they are—in the workplace—motivated them to access the services, especially given their long working hours and the nature of their work. Thus, the MMWC emerged as a more convenient healthcare access method, consistent with findings from another study in Malawi that emphasized the need for men’s outreach health services to improve access [16].

Supervisor support played a critical role in motivating men to access MMWC services. Among men who accessed the MMWC services, having a supportive supervisor encouraged participation. Some supervisors even modeled positive health-seeking behavior by partaking in the services themselves. However, as noted by males who did not access the MMWC services, there was a disparity in the level of support, as not all supervisors accommodated individual employee participation, despite the employer’s support of the clinic’s presence on the premises. Future MMWC initiatives should prioritize sensitization efforts aimed at both employees and supervisors to ensure they understand the benefits of accessing MMWC services and to get necessary buy in.

Furthermore, the study illuminated other implementation challenges impacting equitable access to the MMWC services such as inconvenient clinic hours that resulted in scheduling conflicts, limited awareness due to lack of timely promotion, miscommunication about eligibility, and lack of clear communication about the MMWC’s services and benefits, similar to findings from a systematic review on barriers and motivators of health screening in men [17]. These challenges emphasize the need for employer strategies that go beyond simply offering services. To address these challenges, it is recommended that future MMWC initiatives focus on the vital role supervisors can play in promoting the uptake of HIV testing and other services. This could include workplace policies and practices that involve training supervisors on the benefits of male-friendly healthcare [18]; empowering supervisors to grant flexible work arrangements to participate in HIV testing and other health services; and encouraging open communication about health within the workplace.

Other participants expressed their views with regards to their attitudes for not accessing MMWC services, namely: low risk perception, fear of stigma and discrimination of receiving a positive HIV test results in workplace, and recent negative test results. These findings align with previous studies on barriers to service uptake by men, emphasizing the importance of employers addressing fears related to confidentiality, stigma, and discrimination [7, 19, 20].

Moreover, to promote uptake of HIV testing, investments in demand creation strategies that target employees are equally important. This may involve exploring effective communication methods, educational initiatives, and practical incentives that encourage men to prioritize their health and participate in the MMWCs services [21].

Finally, while an employer initially cited COVID-19 preventive restrictions and workforce disinterest as reasons for not accepting MMWC services, this does not imply that the employer declined the services as the restriction was a temporary measure to contain the epidemic [22]. Employers and employees welcomed the availability of free MMWC services at their workplace and expressed receptiveness to hosting MMWC in the future. This was evidenced by the numerous recommendations provided to improve service uptake to include provision of services separate from women, increasing the number of days and frequency of service provision, sensitization, and incentivizing of HTS and the MMWC; with the latter consistent with literature on incentivizing HIV/Sexually Transmitted Infection (STI) testing uptake, especially testing at a non-clinical setting to modify health behavior [23, 24].

### Study Limitations

This study had some limitations. First, the study was conducted a year after the MMWC services were implemented, which might have influenced the participants’ perceptions experiences, and recall. Next, due to COVID-19 preventive restrictions, we conducted telephone interviews to minimize physical contact. However, this method might have limited the depth of responses we received. In some instances, the interviewers or the participants could not hear each other well, which could have affected the quality of data collected. Additionally, the semi-structured interview guides were not pretested which may have limited the ability to refine questions for clarity and relevance. Future research could benefit from incorporating a pretesting phase to enhance the validity and reliability of the data collected. Lastly, given the focus on a sample size of employees and employers in Blantyre City, Malawi, an urban setting, these findings may not be generalizable of findings to rural settings or other parts of Malawi.

## Conclusion

This study underscores the potential utility of MMWC services, including HIV testing among men. The desire to know their health status, availability of free MMWC services at the workplace, the good quality of services offered, and endorsement of MMWC by supervisors were identified as main motivators to access the MMWC services. Addressing barriers such as work-clinic hours scheduling conflict, lack of adequate promotion, and miscommunication on the criteria about who should attend the wellness clinic are important to facilitate increased MMWC uptake among men in Malawi. Knowing that attitudes and actions of employers and supervisors can influence men’s perceptions of the clinic and their likelihood of utilizing its services, future MMWC initiatives would benefit from sensitizing supervisors and employees about the benefits of the MMWC services, strengthening demand creation, incentivizing both supervisors and employees to increase uptake of HTS and improve health-seeking behaviors.

## Data Availability

No datasets were generated or analysed during the current study.
